# A Layered Trust Information Security Architecture

**DOI:** 10.3390/s141222754

**Published:** 2014-12-01

**Authors:** Robson de Oliveira Albuquerque, Luis Javier García Villalba, Ana Lucila Sandoval Orozco, Fábio Buiati, Tai-Hoon Kim

**Affiliations:** 1 Group of Analysis, Security and Systems (GASS), Department of Software Engineering and Artificial Intelligence (DISIA), Faculty of Information Technology and Computer Science, Office 431, Universidad Complutense de Madrid (UCM), Calle Profesor José García Santesmases, 9, Ciudad Universitaria, 28040 Madrid, Spain; E-Mails: robson@fdi.ucm.es (R.O.A.); asandoval@fdi.ucm.es (A.L.S.O.); 2 Electrical Engineering Department, University of Brasilia, Campus Universitário Darcy Ribeiro, Asa Norte, 70910-900 Brasilia, DF, Brazil; E-Mail: fabio@fdi.ucm.es; 3 School of Engineering and ICT, University of Tasmania, Private Bag 87, Hobart, TAS 7001, Australia; E-Mail: taihoonn@daum.net

**Keywords:** information security, information treatment, risk management, security architecture, trust

## Abstract

Information can be considered the most important asset of any modern organization. Securing this information involves preserving confidentially, integrity and availability, the well-known CIA triad. In addition, information security is a risk management job; the task is to manage the inherent risks of information disclosure. Current information security platforms do not deal with the different facets of information technology. This paper presents a layered trust information security architecture (TISA) and its creation was motivated by the need to consider information and security from different points of view in order to protect it. This paper also extends and discusses security information extensions as a way of helping the CIA triad. Furthermore, this paper suggests information representation and treatment elements, operations and support components that can be integrated to show the various risk sources when dealing with both information and security. An overview of how information is represented and treated nowadays in the technological environment is shown, and the reason why it is so difficult to guarantee security in all aspects of the information pathway is discussed.

## 1. Introduction

Managing information security is critical. Even so, very few formal models are able to secure information. After what happened regarding the information disclosed by Mr. Snowden [1,2], what already seemed very difficult in terms of security, controls, policies, and so on, became much more problematic. Regarding disclosure and technologies that could be used, research on information security has already acknowledged many possibilities, but its level was beyond expectations. The fact is that it is no longer possible to approach information security naively.

The power behind security agencies and information security systems have proven to the world to be something that only security experts were able to understand before. Now, the truth is everywhere, so people can perceive it by themselves. There is a clear shift in the world of security that is taking place after Snowden's information disclosure. As a consequence, information security compliance has been proven to be ineffective.

Having such concerns in mind, up to the present day, discussions about this subject seem endless. Consequently, the information security research field faces new challenges in terms of confidentiality, privacy, anonymity, plausible deniability, and so on.

The world of security is a billion dollar industry [3], only considering cloud solutions. Nevertheless, the problem is that no matter how much money you pay for security, it may never be enough to avoid data theft, information disclosure, system exploitation and other risks that are part of the information technology security context. There is also an understanding that, usually, information security is not properly understood by organizations, because most projects start with no security approach to the problem itself, nor information security related to the whole project.

One way to deal with the information security problem is to manage those risks from different points of view. Risks that can compromise enterprises or governments are associated with natural phenomena, technological risks and human-related risks [4]. Therefore, we propose a suitable and flexible trust information security architecture (TISA) in order to manage risks and security. In summary, the main contributions of this paper are the following: (1) it reviews current information security aspects; (2) it proposes a new-layered trust information security architecture considering several interconnected elements; (3) it introduces a trust layer encompassing all architectural levels; and (4) it proposes information security elements, operations and components for managing risks at different levels considering information treatment.

This paper is organized into the following sections. Section 2 presents relevant reviews and related works in this field. Section 3 presents and discusses the proposed trust information security architecture. Section 4 provides a comparison of our proposal, TISA, with others regarding information security models. Section 5 mentions the information treatment and representation and its connection to the trust information security architecture proposed. Section 6 concludes this paper and points out future works.

## 2. Related Work

A lot has been said about norms, compliance, standards and frameworks regarding information security and the importance of following some of those. Commonly, security standards are guidelines to develop and maintain the information security management system [4–8].

Information security must support business objectives by minimizing risks and developing trust. In addition, it is important to understand that information security requires continuous improvement in a cyclic approach [9]. Standards, such as British Standard 7799 (BS 7799) and ISO 27000 Family [10], are well-known guides in the information security business. Frameworks, such as Information Technology Infrastructure Library (ITIL) and Control Objectives for Information and related Technology (COBIT) [11] are also used in information technology governance in order to help organizations to reduce costs and increase productivity and, in some aspects, aiding security in terms of organization and methodologies [9].

However, ensuring compliance with standards does not guarantee security at all. To deal with information security, it is required to go beyond compliance or the best practices. Up until now and to the best of our knowledge, there is no known proven technology or framework for developing application and information systems without security issues, such as exploitable flaws, configuration errors or system misuse.

Discussions and references precede 1980, considering the information security triad (confidentiality, integrity and availability). The main discussion about confidentiality dates back to 1974, maybe earlier, with Bell and LaPadula [12]. Regarding integrity, the references quote [13] and his model that describes a set of access control rules designed to ensure data integrity. Regarding availability, some references are found in the National Institute of Standards and Technology (NIST) [14]. The exact origins of the ‘CIA triad’ expression appear to be unknown, but apparently, they date back to the NIST publications of the 1990s.

Regarding information security architectures, the zero trust model for cybersecurity [15] conveys a very simple message: Stop trusting packets as if they were people. The idea behind this model is that the concept of internal and external networks should be changed, because one should assume that all network traffic is untrustworthy. In practice, zero trust claims that one should protect internal data from insider abuse as one protects external data in public networks.

In [16], the authors discuss the general effect of risk management, which is limited to security activities regarding an information security architecture on information assets for an organization.

The work proposed in [17] describes an information security architecture with three levels: Domain, component and control. Their target is to align security and business and customer service and manage security, and to create traceability between them, proactively and predictively.

According to [18], it is necessary to develop and improve information security in both administrative and organizational levels. Then, a holistic view of information security based on a survey of the literature, along with a specific Swedish model, is proposed. According to the authors, they have extended the model based on the concepts of semiotic theory and on the perspective of an information system, which included technical, formal and informal sectors.

Behavioral information security shifts the way people are related to information security [19,20]. People have become the center when talking about dealing with information security, a standpoint with which we agree.

Much more has been discussed when it comes to information representation ability. In technology, if information can be retrieved, it is because it has somehow been digitally represented before [21]. Despite the amount of effort, it is still rather difficult to represent and retrieve the correct information for the correct user at the correct moment considering the amount of variables that may be included or related.

The web provides information from a variety of data sources that have great potential for knowledge discovery [22]. One of the long-standing problems is that search engines retrieve a plethora of data; however, most of it is useless, depending on what the user is interested in or searching for. Yet, when it comes to protecting such information during transferences, mechanisms, such as Secure Socket Layer [23] or Transport Layer Security [24], are used very often.

Considering these aspects, in order to protect information, it is very important to understand how it is digitally represented and treated. The users' view may be text, images or both formats. The Internet has redefined the way information is represented and how it is retrieved [25], and as a consequence, the way it is treated has significantly changed. Furthermore, information representation needs extra structural or semantic complements, which transform raw data into something intelligible to people.

Now that we have presented these issues, it is concluded that existing security architectures fail to manage risks, policies, people and assets efficiently. As a contribution, we propose a layered trust information security architecture (TISA) as a way to see how the components are connected regarding information security. In addition, this layered view is important to depict how all the security architecture elements interact with one another.

## 3. TISA: Trust Information Security Architecture

**Figure 1.**
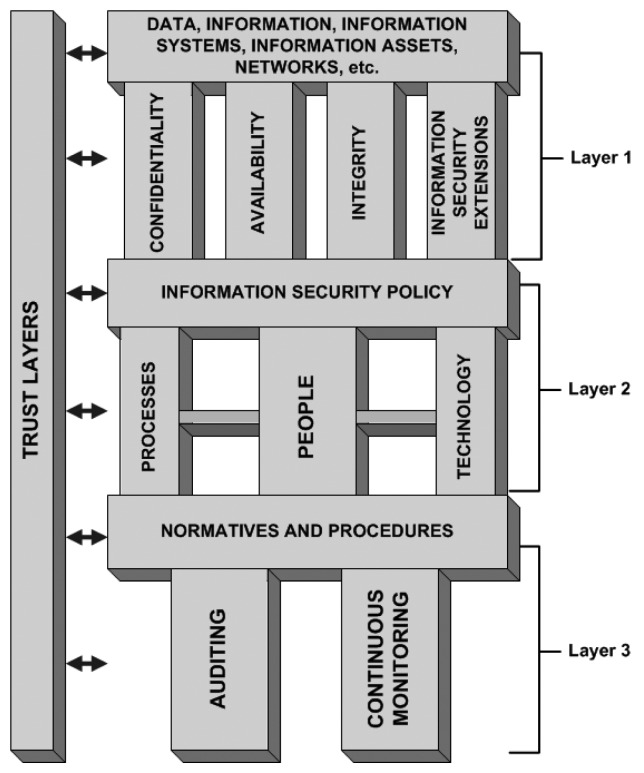
TISA: Layered trust information security architecture.

The way we assess security is based on a layered architecture with components connected in such a way that everything is part of a puzzle that must be well connected and understood, so information security can be seen as a whole. Figure 1 illustrates the proposed information security architecture and its layers.

The following topics describe each layer in a top-down description and its corresponding subtopics, in addition to a brief description of its importance to the puzzle.

### 3.1. Layer 1

This covers the basics to start thinking about information security inside an organization. If one does not understand what data, information, information assets, and so on, are to an organizational environment, there is no point in discussing information security, simply because one does not know what should be protected. It is important to point out that the “protect everything” approach is not effective, and it is very expensive. After that, there is the CIA triad and what we decided to call “information security extensions”. The following items summarize our approach.

#### 3.1.1. Data, Information, Information Systems, Information Assets, Networks, *etc*

Generally, this is the layer in which data is important to organizations or in which individuals are found or mapped. Considering the importance of data for organizations nowadays, information can be retrieved from data and information systems. Using the correct tools and techniques, it is possible to create new knowledge from data, which, at first, made no sense at all. In the vast data world, crawling, retrieving, analyzing, discovering relations and finding hidden patterns are the areas in which efforts have been made in order to create knowledge from raw data.

When it comes to information assets, it is also very important that they are identified and labeled, and the relationship to information should be clearly understood by the organization and the ones responsible for its protection.

Networks connect every piece of data, information and information assets, so that anyone with granted access is able to browse them. When it comes to information or data in transit, it is important to remember that, in networks, bits are not treacherous by themselves: People who control them or how they do so (process, hardware or software) are who/what makes them dangerous or not. Therefore, marking network perimeters, having network policies, providing defense mechanisms *etc*., still are ways of managing what comes in and out of a network.

The proper use of such mechanisms is the key to understanding what happens in the network and information systems. These components of the puzzle create inputs for supporting decisions related to information security.

#### 3.1.2. Confidentiality

Confidentiality is the information security property responsible for preventing unauthorized disclosure of information. In other words, it is a mechanism to give access to authorized individuals or systems in the organization only. It applies principles, such as the “need-to-know”, and in order to be effective, confidentiality must ensure that access to vital information is limited only to those who specifically need to have access to or use that particular information. This piece of the puzzle deals with the organization's ability to keep its data, information and knowledge protected from unauthorized disclosure.

#### 3.1.3. Availability

Every piece of information has a specific value or use depending on a specific end. In order for information systems to serve their purpose, information must be available when necessary. To acquire such a property, all information systems, networks, databases, information assets, storage mechanisms, and so on, must be accessible by the ones who have granted access to manage them when required. Information is unavailable, not only when it is lost or destroyed, but also when its access is denied or delayed for those who are authorized to use it.

Therefore, to guarantee availability, communication channels used to access information, wherever it is, must be operating correctly and according to information security policies. This piece of the puzzle deals with the required organizational skills to guarantee that the ones who have the necessary permissions regarding its use, when necessary, can access information.

#### 3.1.4. Integrity

Integrity is the ability to guarantee the accuracy and consistency of data and information during its entire life cycle. Considering such a property, data or information should not be unauthorizedly destroyed or modified, which would hinder its detection. Integrity has to guarantee that the information is accurate, reliable and, most importantly, that it has not been changed or tampered with by an unauthorized party all of the sudden. Integrity may include the ability to verify whether information content has been unauthorizedly altered, to determine the origin of any action in the system and to associate it with a specific entity. This piece of the puzzle deals with the way information is securely managed in organizations, with no loss of its basic properties.

#### 3.1.5. Information Security Extensions

There is discussion among the information security experts regarding whether the information security triad is enough to stand as the basis of information security by itself; we believe it is not. However, it is also very difficult to extend all types of information security properties or attributes, thus ensuring that your information is secure for you to have access to the so-called extensions.

We picture information security extensions as a group of new attributes or properties to protect information and systems, but that are not limited to it. We summarizes some of them from this work's perspective.

- Authentication: This is the measure that verifies identities. In a communication process, the party must provide evidence that it is the person or entity to whom the credentials belong. At least one identification element is included in the authentication process. Usually, this process requires personal or individual knowledge, ownership information or personal information, despite the way these are linked. They are related to something you know, something you have or possess and some information about yourself.
- Access control: This is what restricts access to a certain datum, information or resource. According to this principle, once access control is guaranteed, the entity should be able to extract, enter or use a particular piece of information or an information system. Generally, access control mechanisms begin with the identification and authentication processes.
- Non-repudiation: In short, a party within a communication process cannot deny having received specific information, nor can the other party deny having provided specific information. Generally, cryptographic systems can support non-repudiation efforts using digital signatures, but the discussion goes beyond the technological field, because one cannot guarantee that such a signature proves authenticity and integrity, thus preventing repudiation; for instance, cases of data theft. The opposite is so-called plausible deniability, in which one's culpability might be denied or mitigated or it is even possible to deny that one was responsible for it in the first place.
- Authenticity: This is a way to ensure that data, systems, communication processes or information are genuine. Authenticity is also responsible for validating that both parties involved in a communication process are who they claim to be.
- Privacy: This regards the right to control information about an individual and the right to limit access to that information. It is also related to domains in which individuals have the right to keep confidential information and data and to share them in private conversation [25]. It is also the right to protect your personal information and to prevent invasions of privacy. Privacy is preserved for one's good [26].
- Anonymity: This is simply a result of not having identifying characteristics disclosed or made available to the public, which would allow the identification of an entity. In a communication system, anonymity is the party's state of being anonymous and, yet, being able to respond to or interact with another party (without revealing its identity). It is also related to terms, such as untraceability and unlinkability [27].
- Authorization: This is the process of providing access to particular information or a system to a party based on their identity. After going through the authorization process, one is allowed to have access to some or all of the data in a specific environment or system. Authorization allows an entity to access and perform determined actions regarding data. In order to be effective, authorization should be based on the roles that an entity may have.

Accordingly, these properties are able to support themselves as elements that depend on many other complex characteristics, such as context, technologies that support them, usage objectives, and so on. In short, information security extensions are a bit of the puzzle that completes the information security basis, and in addition to that, some of the terms are sufficient by themselves.

### 3.2. Layer 2

According to the top-down description, this is the layer where the architecture will help to understand how, why and which technology may be used and who may use it in order to provide information security for higher levels. The following items summarize these.

#### 3.2.1. Information Security Policy

The information security policy is a high-level document that outlines specific requirements or rules that must be met regarding information security. Generally, this policy is very specific and covers only one organization. The information security policy also links security management to security issues and security controls. Once the information security policy is straightforwardly attached to the organization, it is important that the policy is formulated taking the organization's features into consideration.

The information security policy should explain the fact that all users need information security within the organization and should complement business objectives. Thus, the policy should be aligned with the organization's strategic information plan [28].

The information security policy is the piece of the puzzle that guides people during the creation of the processes and the definition of technological components in order to help to protect information. Daily practices show that without information security policies, things are done based on individual efforts that, usually, are non-effective.

#### 3.2.2. Processes

Inside information security, processes are formal mechanisms to identify, measure, manage and control risks related to information or its value to the organization. Processes are very important when information security is in evidence, thus it should not be seen as a black box or something that is meaningless to the organization. To be more specific, processes include formal and informal mechanisms (large and small, simple and complex) and provide a fundamental link to all dynamic interconnections.

Processes derive from strategies and implement the operational side of what should be done within the organization. To be useful and provide advantages to the enterprise, processes must be directly linked to business requirements and be aligned with information security policy in the puzzle. They also should consider emergency situations and be adaptable to changes in requirements. It is important that information security processes are well documented and communicated to human resources, which should know about them. It is fundamental that processes should be reviewed periodically to ensure efficiency and effectiveness [28].

#### 3.2.3. People

“People” is the number one subtopic of the puzzle and represents human resources. In general, people develop and implement each part of an information security policy, create and maintain processes, information assets, define which technology should be used, design networks, *etc*. Security issues affect people and their relationships, values and behavior. When working with information security, it is important to address points, such as strategies related to hiring, access, responsibilities, training, dismissal, damage and whatever is considered important to be addressed, to help to maintain the organization's information security strategy.

When dealing with people, it is very important to understand that what sounds obvious to security experts definitely does not sound obvious to someone without the same experience level. People are the ones whose actions within the organization influence the information security triad and security extensions related to data, information, information systems, information assets, network usage, resources and whatever is valuable to the organization. In short, individuals' actions and motivations have direct positive or negative influence on information security. All of this is straightforwardly related to behavioral information security [19,20].

#### 3.2.4. Technology

This the piece of the puzzle that is the set of all informatics systems, applications, tools, infrastructure and defense mechanisms that the organization applies to achieve its goals and to help information security. Technological elements may frequently change and update, become obsolete very fast or be the core of an organization's infrastructure. Technology may also solve security threats and risks. Users and the organization's environment also have a strong impact, once technology can be perceived as a way to avoid security controls being transpassed [28].

It is very important to remember that technology, by itself, does nothing. It should be seen as part of a complex system that has specific needs to protect what is valuable within the organization and should work along with people and processes in a cyclic pattern, all these guided by information security policies.

### 3.3. Layer 3

This represents the piece of the puzzle that deals with daily activities and how they should be done. In addition to that, it is related to what actions should be taken in case a specific problem arises. Bear in mind that complex systems and their security practices are guides to keep information secure; but norms, procedures, monitoring and auditing will give systems administrators the tools to help them keep information, information assets, networks, systems, *etc*., more protected.

#### 3.3.1. Normatives and Procedures

Basically, a normative is related to how things should be and how they should be rated. It is also related to how to classify things as good or bad or which actions are right or wrong. Normatives are fundamental for prioritizing goals, organizing and planning actions in order to define how things should be done. Considering the user's perspective, it is much more common than it may seem, but users generally try to follow social expectations rather than following security procedures [29]. This is not desirable regarding security perspectives, because such conflicts lead to security policy inconsistency.

In general, normatives can be implicit or explicit, while security policies are explicitly mandatory normatives. Procedures are step-by-step definitions of how things must be done considering specific contexts. It is also a guide to what one should do if a particular condition is met.

A procedure is way to provide minimum safeguards (administrative, technical, physical or all of these) that are employed to protect sensitive information. It is expected that procedures are technical and intended for information custodians, systems administrators and information technology personnel within the organization.

#### 3.3.2. Auditing

Consider that auditing information security is a process that takes measures in terms of qualitative and quantitative assessing of the current state of what is being audited regarding specific criteria of information security.

Auditing is the key to discover risks, technical flaws, policies, procedures and normative problems. Bearing in mind that auditing is an everlasting process, when auditing information security, one should be prepared to cover topics from the physical security of data centers to logical security, including network perimeters, system configuration and information systems. Each one has its peculiarity, thus different methods for auditing should be taken into consideration and should also be applied to guarantee the auditing results.

Always assume that information security auditing is for information security professionals and the entire auditing process should be part of an overall plan. Auditing will provide at least an independent review of the adequacy and effectiveness of the internal controls regarding business process, people behavior, infrastructure current state, policies, normatives and procedure compliance.

Auditing is also an operating measure to verify the state of what is being audited in a specific period of time, so it can verify previous behavior, but will not guide the organization for all future possibilities.

#### 3.3.3. Continuous Monitoring

Continuous monitoring keeps track of ongoing knowledge of information security, vulnerabilities, threats [30] and, thus, their associated risks. It is the key to support decisions regarding risk management. It also guides decisions regarding protective and proactive measures.

This piece of the puzzle is important, because compliance with international normatives and standards does not guarantee that the organization's security objectives will be achieved. In practice, using normatives and standards will help to achieve a higher level of maturity regarding information security.

Monitoring systems begin with the definition of what, how, when and why information assets or any part of the architecture should be monitored. It relies on technology, processes, procedures, operating environments and people. It is deeply connected to the understanding of organizational risk tolerance. It also helps to set priorities and to consistently manage risk inside the organization. It should be standardized, so that one is able to give visibility and identify security status at all monitored information assets.

The task of monitoring can be perceived as an active security component [31]. It can sense the infrastructure's current state. It also considers a wide variety of data and information in order to react according to specific defined policies as a way to help protect the information within the organization. Continuous monitoring should be based on measures, such as protection, sense, adjustment, collection, alarms and others related to information security.

### 3.4. Trust Layer

When it comes to security, trust is zero or one. You trust your information systems, network, *etc*, or you do not; “maybe” should be avoided. Usually, trust can be acquired by empiric observation, by formal proof of the systems and the mechanism involved and other techniques [32]. Once all expectations are fulfilled, one may establish trust. The problem is that trust by its own means is an expectation. It is a probability that things will work and keep working as they are supposed to.

Considering a security perspective, for something to be trusted, it must be clearly identified and operate exactly as planned and expected. It also must not do anything it was not supposed to do and must be able to operate nonstop. If a trust approach is taken, and it is acknowledged that a system may be or has been compromised, it is enough to make it suspicious or untrustworthy.

Trust and security are closely related [32]. If security objectives are considered, it is clear that trust is connected to security, because information security depends on people and security extensions, such as authentication, authorization, access control, non-repudiation, *etc*. However, in practice, information security approaches should be “trust, but verify.” [15]. Additionally, verify always, even if apparently there is nothing suspicious.

In general, trust is the piece of the puzzle in which there is enough knowledge about information, systems, technology and other components to help one to make assertions, such as “fully secured” or “information is secure because a particular condition was met”. Such cases are very common nowadays, because most people do not build information systems. They use them and trust them in terms of functionality and security. However, the scope of trust goes beyond that. It is more related to the ability that one may have to check all of the particularities of the software or hardware he uses and, also, being able to careful choose what he trusts or not. Additionally, he should define what would be the security acceptance regarding a particular hardware, software or information system.

Having such particularities in mind, the layered approach herein presented allows one to see that trust is connected as part of the security architecture and may be addressed when needed in all layers and in all components that are part of the architecture.

The IEEE Cybersecurity Initiative published a list of what they felt were the top security design flaws [33], where trust is considered as an important subject when security is considered. They state, for example, that data sent to an information system by untrusted clients or channels should be assumed to be compromised until proven otherwise. In such cases, if the integrity cannot be verified, the data inherently are not trustable.

In short, the main message is to make sure all data received from an untrusted client or system are properly validated before processing, because one may be exposed unnecessarily to vulnerabilities by trusting components that did not earn that trust [33].

### 3.5. Measuring Trust in a Distributed Environment

In [34], the authors present a model able to measure trust regarding groups in distributed environments. As a particular result, using the same group trust model as in [34], we extended the measure capabilities with more nodes in a simulation.

In these results, the test bed is composed of five different groups with 200 nodes in each group, having a total amount of 1000 nodes. In all of the result graphs, the x-axis represents the number of rounds that the nodes perform for their particular job, having a total of 20 rounds used to calculate group trust. The y-axis represents the amount of trust calculated in each round using the model proposed in [34]. We simulated three different scenarios.

**Figure 2.**
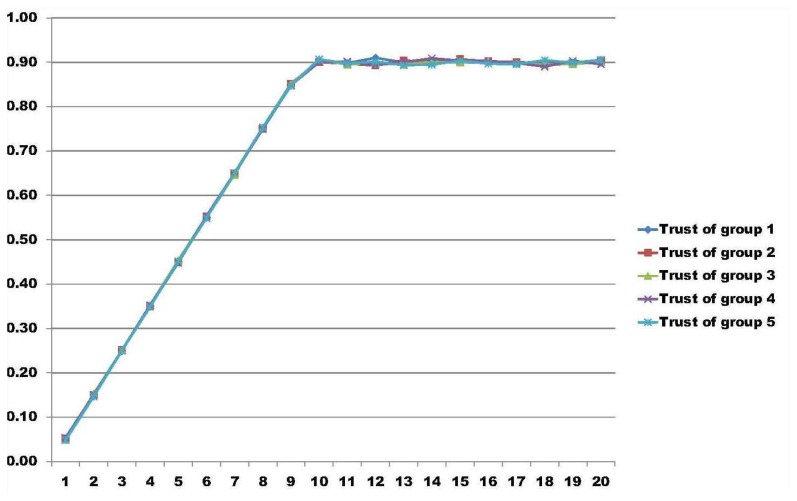
Ideal scenario where all groups are trustworthy.

In the first scenario, we simulate the ideal environment, where all nodes behave accordingly and perform their job as expected, so that they fulfill the other nodes' outlooks. In this case, this represents a scenario where there are no malicious nodes inside the network and all groups are considered trustworthy. Figure 2 illustrates this perspective.

**Figure 3.**
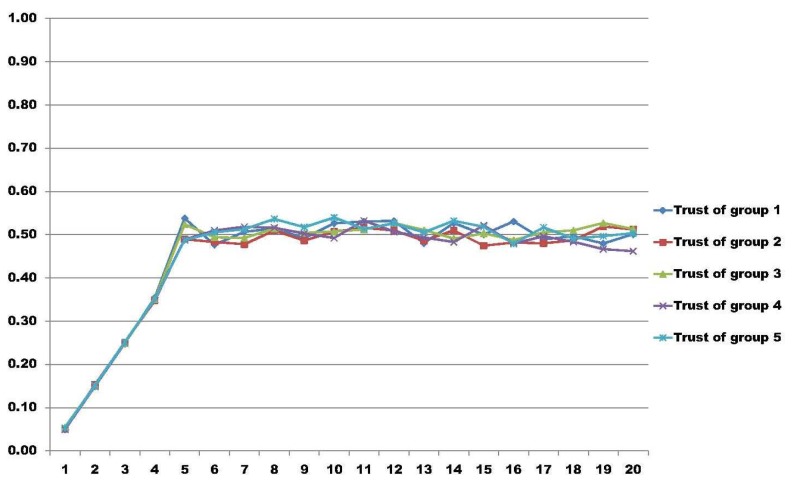
Worst scenario where all nodes in all groups behave randomly.

In the second scenario, we have all nodes moving in a random way. This scenario, illustrated in Figure 3, represents the worst environment possible regarding group trust. In a condition like this, it is not possible to say if the node is bad or good, because it changes its behavior unexpectedly. In such cases, one particular node may be trustworthy to one entity, because it may fulfill one's expectation in a particular condition, but next, it may completely change its state.

**Figure 4.**
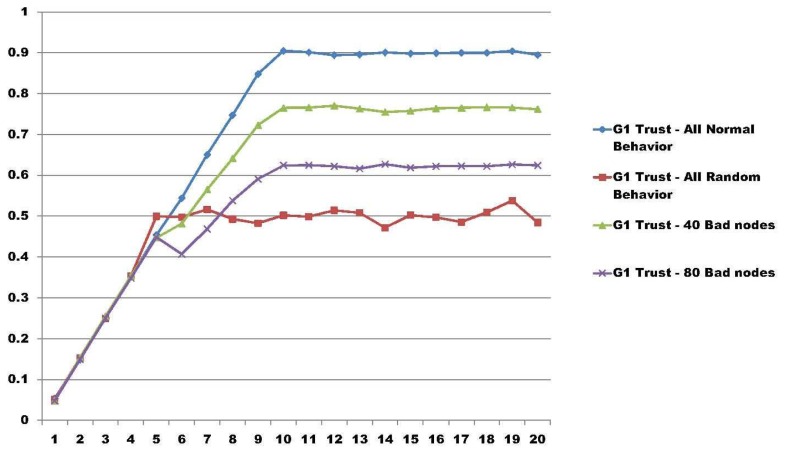
Group trust view for the first group.

The last scenario, illustrated in Figure 4, summarizes the view of Group 1 in four different approaches. It compares Group 1 trust with all nodes behaving accordingly and all nodes behaving randomly. Then, it shows a particular case where 40 nodes (20% of the members of Group 1) change their behavior after Round 5. After, it shows 80 nodes (40%) of the members with changes in their behavior. In both cases, with 40 and 80 bad nodes, it is possible to see that when a coalition of nodes are made, the group trust model is able to address the change in the trust of the group.

## 4. Comparing Information Security Architectures

It is very hard and also complex to compare information security architecture in general. There is no common approach or benchmark able to measure every peculiarity of each known architecture. Additionally, doing so, this still may be incomplete for addressing every aspect related to information security.

The work in [35] addresses the problem of how hard it is to compare information security models. In general, it recommends to first perform a high-level comparison based on the criteria definition. Then, it addresses that it is also important to create an appropriate selection of candidates and then perform a more time-consuming extensive evaluation.

The authors in [36] review various information security standards and compare major information security standards ISO 27001, BS 7799, Payment Card Industry Data Security Standard (PCIDSS), ITIL and COBIT in terms of information security policy, communications and operations management, access control, information system acquisition, development and maintenance, organization of information security, asset management, information security incident management, business continuity management, human resources security, physical environment security and compliance. The authors conclude that each standard plays its own role and position regarding information security management systems.

The work in [37] also reviews some information security standards and architectures in terms of security policy theory, risk management theory, control and auditing theory, management system theory and contingency theory.

Having the assumptions of previous works in mind and taking a trust approach, we compare TISA with two major widely known information security guides. Table 1 summarizes the comparison approach.

As seen, basically, TISA considers trust as an important role regarding information security, while ISO 27001 and BS 7799 have little or no consideration of trust. Furthermore, TISA considers that compliance guarantees no security. Compliance in this case helps increase the level of maturity regarding information security.

## 5. Information Representation and Treatment and Their Relation to TISA

When it comes to security, the type of information, that operations that can be performed regarding it and the supporting components must be considered if you want to protect them. In fact, you cannot protect information if you do not understand where it is stored, how it is represented or who or what manages it. In other words, how information is digitally treated is key to trying to protect it.

**Figure 5.**
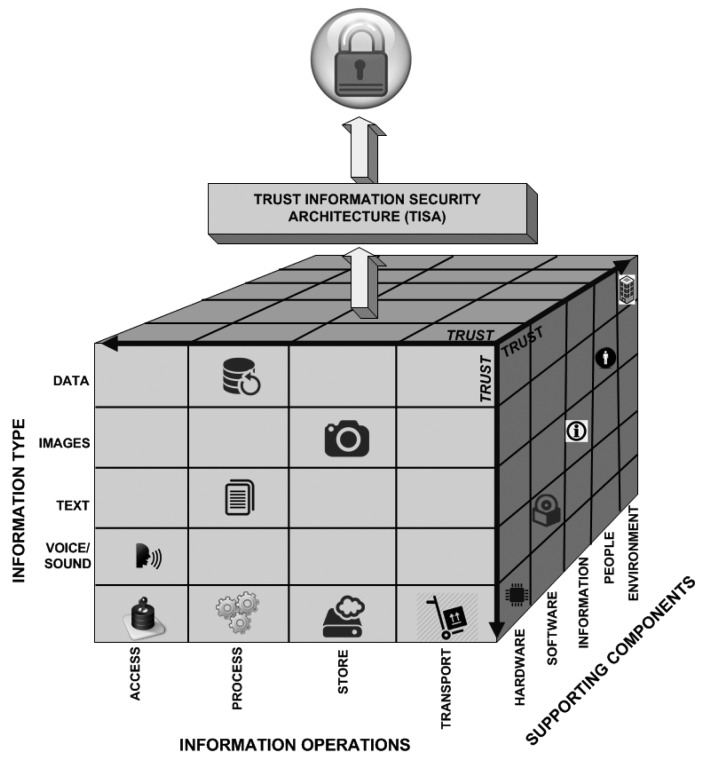
Types of information, operations and components.

Figure 5 depicts how information is treated regarding its basic representation, the basic operations to process it and where these activities may digitally happen from a cyber perspective. If the whole complexity of Figure 5 is considered, it sounds very difficult to guarantee or to achieve information security. From our perspective, a trust layer is used as a means to explain when technology or a regular approach by itself is not enough to guarantee security during the entire process. For instance, from the users' perspective, things should work just as when such things are supported by an information security architecture, as described in previous sections.

Considering the digital representation of information, the types of information can be images, text, voice or sound and videos (a combination of images and sound). When it has no particular characteristic, it can be perceived as data (which may be a set of digital representations, such as database files or proprietary data types).

Once the information has its representation regarding a particular information type or a combination of them, it may undergo specific operations in the digital environment. Operations, such as access data, process text, store voice records or transport any of those from one place to another using any kind of media (disks, cell phones, tablets, networks, *etc*.), are very common in modern organizations. Any combination of such information operations is also possible; for example, one particular user may wish to transform a text file into a voice file, or *vice versa*, or a particular user wishes to describe an image giving it more sense then the previous one. All of these processes are possible and very common in the digital environment, and there are a lot of possibilities when it comes to the combination of information type and information operations. Thus, to protect it in all existing scenarios is rather difficult.

The supporting components are where or how the information is used. For example, a video is stored in hardware and uses proprietary software to play. This is also where people directly act regarding information. For example, if a user copies a file from the database to a thumb drive disk through the network, different supporting mechanisms are applied so that the user is able to achieve his or her goal.

The entire cube depicts how information is digitally represented and treated. Therefore, it should be considered that information security should be guaranteed inside environments where the following activities take place: copying files, printing images, writing data to database systems, using distributed network environments, network connections of all types are supported, users making common mistakes, such following unknown hyperlinks, and so on.

According to a very simple approach, it is a complex matrix of at least three dimensions to keep one piece of information secure inside such environments. That happens because one should consider the type of information, which operations it may undergo and what information support is used, all that just as a small example of to keeping information secure.

In our perspective, we believe it to be very difficult to achieve information security regarding the cube perspective depicted in Figure 5 without using an information security architecture as proposed in Section 3. According to [1,2,15], using technology or being compliant with standards is not enough to keep information protected. More needs to be accomplished, and in such cases, trust should be used even without knowing it in first place.

## 6. Conclusion and Future Work

Information security is not an end by itself; it is a means to achieve an end [5]. Information security is also a constantly developing subject, due to the ever-increasing scale and complexity of security threats in recent days.

Nowadays, the information security research field is becoming more and more important, significantly because the world is highly interconnected, with the use of networks to carry critical and sensitive information. Considering a cyberspace perspective, information may be in use by an entity, may be stored inside media or may be in transit through some communication channel. Additionally, if you consider your information important and care about information security, you should protect it in whatever state it is.

All things considered, at the present time, one cannot protect his/her information without understanding its whole life cycle. Bearing in mind a deeper perspective, if information security is a goal, one should be able to represent it, process it and use it in a cyberspace environment where people, technology, information assets, hardware, software, and so on, are amply used and connected. Consequently, security measures should be taken to guarantee protection, and this is where the information security architecture comes in, because the “protect one piece of information” scenario by itself has been proven to be ineffective [1,2].

In this paper, we introduced a layered trust information security architecture (TISA) and its connected elements, which are useful to manage risks at different levels regarding information in an organization. In the layered architecture, every sub-item is a piece of the puzzle, so information security can be analyzed as a whole.

Government, organizations and enterprises consider that information security management needs a systematic approach to consistently address security in every layer, reducing unmanaged risks and improving operational security efficiency. From such a perspective, the proposed layered information security architecture can be used as a guide to helps achieve better results regarding information protection.

The proposed architecture provides several opportunities for further security research. Regarding future works, first of all, we intend to extend the trust functionalities and better explain the relations between the sections and all layers of the architecture. Secondly, we intend to carry out more research to address all of the details regarding trust in the architecture. Thirdly, we intend to detail how the pieces of the puzzle, such as people, technology and processes, should be connected and guided using information security polices, as in [28]. Finally, the information representation and treatment cube can be improved regarding an information security perspective that would connect it to an information security life cycle.

## Figures and Tables

**Table 1.** Comparison criteria.

**Architecture\Criteria**	**ISO 27001**	**BS 7999**	**TISA**
Trust as an important role	No	No	Yes
Address security extensions	Yes	Yes	Yes
People as an important role in security	Yes	Yes	Yes
Continuous monitoring activities	Yes	Yes	Yes
Auditing activities	Yes	Yes	Yes
Address security compliance	Yes	Yes	No

## References

[1] Greenwald G., MacAskill E., Poitras L. (2013). Edward Snowden: The whistleblower behind the NSA surveillance revelations. The Guardian.

[2] Richelson J.T. (2013). The Snowden Affair. Washington Post.

[3] Release G.P. (2013). Gartner Says Cloud-Based Security Services Market to Reach 2.1 Billion in 2013.

[4] Blakley B., McDermott E., Geer D. Information security is information risk management.

[5] Peltier T.R. (2013). Information Security Fundamentals.

[6] Disterer G. (2013). ISO/IEC 27000, 27001 and 27002 for Information Security Management. J. Inf. Secur..

[7] Stamp M. (2011). Information Security: Principles and Practice.

[8] Posthumus S., von Solms R. (2004). A framework for the governance of information security. Comput. Secur..

[9] Secure Your Information: Information Security Principles for Enterprise Architecture. http://www.tisn.gov.au/Documents/Secure_Your+Information+-+Information+Security+Principles+for+Enterprise+Architecture+-+Report.pdf.

[10] Whitman M., Mattord H. (2013). Management of Information Security.

[11] Parvizi R., Oghbaei F., Khayami S.R. Using COBIT and ITIL frameworks to establish the alignment of business and IT organizations as one of the critical success factors in ERP implementation.

[12] Bell D.E., LaPadula L.J. (1973). Secure Computer Systems: Mathematical Foundations.

[13] Biba K.J. (1977). Integrity Considerations for Secure Computer Systems.

[14] Burrows J.H. (1983). Guideline for Computer Security Certification and Accreditation.

[15] Contreras J.L. (2013). Developing a Framework to Improve Critical Infrastructure Cybersecurity.

[16] Jeong G.H., Yi D.W., Jeong S.R. (2010). The Effect of Composition and Security Activities for Information Security Architecture on Information Asset Protection and Organizational Performance. KIPS Trans. Part D.

[17] Tang C.L. (2014). Establish a Dynamic Business Driven Integrative Information Security Architecture. Appl. Mech. Mater..

[18] Åhlfeldt R.M., Spagnoletti P., Sindre G. (2007). Improving the Information Security Model by Using TFI. New Approaches for Security, Privacy and Trust in Complex Environments.

[19] Crossler R.E., Johnston A.C., Lowry P.B., Hu Q., Warkentin M., Baskerville R. (2013). Future directions for behavioral information security research. Comput. Secur..

[20] Aurigemma S., Panko R. A composite framework for behavioral compliance with information security policies.

[21] Chu H. (2003). Information Representation and Retrieval in the Digital Age.

[22] Nayak R., Senellart P., Suchanek F.M., Varde A.S. (2013). Discovering interesting information with advances in web technology. ACM SIGKDD Explor. Newsl..

[23] Freier A., Karlton P., Kocher P. (2011). The Secure Sockets layer (SSL) Protocol Version 3.0.

[24] Dierks T. (2008). The Transport Layer Security (TLS) Protocol Version 1.2.

[25] Diffie W., Landau S.E. (2007). Privacy on the Line: The Politics of Wiretapping and Encryption.

[26] Anderson R. (2008). Security Engineering.

[27] Hughes D., Shmatikov V. (2004). Information hiding, anonymity and privacy: A modular approach. J. Comput. Secur..

[28] An Introduction to the Business Model for Information Security. http://www.isaca.org/Knowledge-Center/BMIS/Documents/IntrotoBMIS.pdf.

[29] Pieters W., Coles-Kemp L. Reducing normative conflicts in information security.

[30] Dempsey K., Chawla N.S., Johnson A., Johnston R., Jones A.C., Orebaugh A., Scholl M., Stine K. (2011). Information Security Continuous Monitoring (ISCM) for Federal Systems and Organisations.

[31] Hand R., Ton M., Keller E. Active security.

[32] Lamsal P. (2001). Understanding Trust and Security.

[33] Avoiding the Top 10 Security Flaws, 2014. http://cybersecurity.ieee.org/center-for-secure-design/avoiding-the-top-10-security-flaws.html.

[34] De Oliveira Albuquerque R., García-Villalba L.J., Kim T.H. (2014). GTrust: Group Extension for Trust Models in Distributed Systems. IJDSN.

[35] Van Os R. (2014). Comparing Security Architectures: Defining and Testing a Model for Evaluating and Categorising Security Architecture Frameworks. Master's Thesis.

[36] Susanto H., Almunawar M.N., Tuan Y.C. (2011). Information security management system standards: A comparative study of the big five. Int. J. Electr. Comput. Sci. IJECS-IJENS.

[37] Hong K.S., Chi Y.P., Chao L.R., Tang J.H. (2003). An integrated system theory of information security management. Inf. Manag. Comput. Secur..

